# Polylactide Composite Pins Reinforced with Bioresorbable Continuous Glass Fibers Demonstrating Bone-like Apatite Formation and Spiral Delamination Degradation

**DOI:** 10.3390/polym11050812

**Published:** 2019-05-06

**Authors:** Xiao-Yan Cao, Na Tian, Xiang Dong, Cheng-Kung Cheng

**Affiliations:** 1 School of Biological Science and Medical Engineering, Beihang University, Beijing 100083, China; caoxiaoyan042@163.com or caoxiaoyan@buaa.edu.cn; 2 Beijing Engineering Laboratory of Functional Medical Materials and Devices, Beijing Naton Technology Group Co. LTD, Beijing 100094, China; tianna@naton.cn (N.T.); dongxiang@naton.cn (X.D.); 3 Beijing Advanced Innovation Center for Biomedical Engineering, Beihang University, Beijing 100083, China

**Keywords:** degradation behavior, spiral delamination, bone-like apatite, bioresorbable continuous glass fibers, polylactide composite pin

## Abstract

The emergence of polylactide composites reinforced with bioresorbable silicate glass fibers has allowed for the long-term success of biodegradable polymers in load-bearing orthopedic applications. However, few studies have reported on the degradation behavior and bioactivity of such biocomposites. The aim of this work was to investigate the degradation behavior and in vitro bioactivity of a novel biocomposite pin composed of bioresorbable continuous glass fibers and poly-L-D-lactide in simulated body fluid for 78 weeks. As the materials degraded, periodic spiral delamination formed microtubes and funnel-shaped structures in the biocomposite pins. It was speculated that the direction of degradation, from both ends towards the middle of the fibers and from the surface through to the bulk of the polymer matrix, could facilitate bone healing. Following immersion in simulated body fluid, a bone-like apatite layer formed on the biocomposite pins which had a similar composition and structure to natural bone. The sheet- and needle-like apatite nanostructure was doped with sodium, magnesium, and carbonate ions, which acted to lower the Ca/P atomic ratio to less than the stoichiometric apatite and presented a calcium-deficient apatite with low crystallinity. These findings demonstrated the bioactivity of the new biocomposite pins in vitro and their excellent potential for load-bearing applications.

## 1. Introduction

Human bone is a stiff and lightweight composite material with a nanocomposite structure derived from mineralized osseous tissue composed of carbonate hydroxyapatite and collagen [1,2,3]. Previous studies [2,3,4,5,6,7,8,9,10,11,12] introduced biomimetic composites for orthopedic applications which used biodegradable polymers blended with β-tricalcium phosphate (TCP), hydroxyapatite (HA), or bioactive glass. Although the addition of these bioceramics improved the biological properties of the composites, including osteoconductivity [4,13] and/or osteoinductivity [10,14], the relatively low overall mechanical strength and modulus still resulted in unsatisfactory healing, particularly when used for load-bearing orthopedic applications [15]. 

More recently, totally bioresorbable composites consisting of silicate glass fibers in a biodegradable polylactide matrix have been introduced as novel orthopedic materials due to their advanced mechanical properties which can support the bone during healing and also gradually degrade over time, allowing the bone to regain its natural strength [1,16]. The mechanical behavior of such composites reinforced with silicate glass fibers [1] may be more suitable for load-bearing applications than similar polylactide composites reinforced with phosphate glass fibers [17,18] or 13–93 bioglass fibers [3], and poly(butylene succinate) reinforced with basalt fibers [19]. Any successful resorbable orthopedic material should have sufficient mechanical properties to support the fracture, a degradation behavior matched to the healing rate of bone, and advanced bioactivity to promote the generation of new bone [20]. 

Lehtonen et al. [1] demonstrated that the degradation behavior of poly-_L_-co-_D, L_-lactide (PL/DLLA) composites in vitro can be tailored by changing the oxide composition of the silicate glass fibers. It has also been shown that the dissolution behavior of silicate glass fibers in an aqueous medium can be easily adjusted by simply modifying the glass composition [16]. Previous studies [1,16] have mainly focused on varying the properties of the silicate glass fibers, but the composition of the polylactide matrix can also significantly affect the degradation behavior [21]. Poly-_D, L_-lactide (PDLLA) materials with high mechanical performance have been reported to degrade after approximately 1–1.5 years, in comparison with the much longer degradation rate of 5–6 years for conventional poly-_L_-lactide (PLLA) [21]. The degradation behavior and bioactivity of implants may also be affected by other factors [1,5,15,22] such as the use of long fiber reinforcement [1,22] and the degradation environment [5]. However, little information is available on the mode of degradation and the bioactivity of such novel biocomposites.

Understanding an implant’s ability to form apatite in simulated body fluid is useful for evaluating its ability to bond bone in vivo [23]. Various materials have been shown to bond to living bone through a layer of apatite [22,23,24], and this apatite layer can be reproduced on the surfaces in an acellular and protein-free simulated body fluid (SBF) with ion concentrations nearly equal to those of human blood plasma, meaning the apatite thus formed is similar to the bone mineral in its composition and structure. The osteoconductivity of several different biodegradable composites of polymers and bioactive glass has also been demonstrated by the ability to form apatite in vitro [8,14,25]. 

Owing to the complicated process whereby an implant is resorbed and loading is gradually transferred back to the host tissue, an appropriate preliminary in vitro study is required to understand the properties of the implant and how the materials behave. Therefore, the objective of this study was to investigate the degradation mechanism and apatite formation of novel biocomposite pins made of poly-_L_-co-_D_-lactide (PLDLA) reinforced with bioresorbable continuous glass fibers in simulated body fluid for 78 weeks. The long-term degradation behavior of the biocomposite pins was examined and the in vitro bioactivity was also studied to evaluate the feasibility for load-bearing orthopedic applications.

## 2. Materials and Methods 

### 2.1. Biocomposite Pins

Biocomposite pins were manufactured from 72.5% poly-_L_-co-_D_-lactide (PLDLA, L/D = 80/20) biocomposite billets reinforced with 27.5% bioresorbable continuous glass fibers (CGFs). All billets were supplied by Arctic Biomaterials Oy Ltd. (ABM), Finland. The CGFs are referred to as ‘X3 fibers’ by ABM, and had a mean diameter of 15 μm. Pure PLDLA pins were also created as a control group. All pins were machined from the rod billets to be 50 mm in length and 2 mm in diameter. Seventy-two pins were created for each group. All pins were sterilized by ethylene oxide (EtO) prior to use.

### 2.2. Soaking Medium

An SBF medium was prepared according to ISO 23317 (Implants for surgery—In vitro evaluation for apatite - forming ability of implant materials) and closely matched the ion composition of human blood plasma (Table 1). The SBF was used to investigate the in vitro bioactivity and degradation behavior of the biocomposite pins over time. The pins were fully immersed in the SBF at the beginning of the experiment, and then a sample number of pins was removed at set intervals to test the properties. The pH of the solution was adjusted to 7.40 ± 0.2 with 1M HCl at 37.0 °C. Ultrapure water with a resistivity of 18.2 MΩ·cm at 25 °C was used.

### 2.3. In Vitro Degradation 

All pins were immersed in the SBF (pH = 7.4 ± 0.2) in sealed plastic containers with a smooth inner surface at 37 ± 1°C. The volume of the SBF for testing was calculated as the ratio of the SBF volume to the apparent surface area of the pin, and the volume was adjusted as necessary to achieve a ratio of 100:1. The SBF was changed every two weeks to inhibit clouding of the soaking medium, indicating microorganism contamination. The pH and temperature of the solution were measured in at least two different containers chosen randomly at each test time point. The test intervals were 0, 0.14 (24 h), 3, 6, 9, 12, 15, 18, 26, 39, 52, and 78 weeks. At each time point, six biocomposite pins from each group (reinforced PLDLA pins and pure PLDLA pins) were removed from the solution and tested: three samples for testing molecular weight and three samples for checking changes in mass, size, thermal properties, micromorphology, and microstructure. Changes in the pH level of the buffer solution were also analyzed. All samples were dried in a vacuum oven at room temperature until their weight loss reduced to a negligible level. All results are given as a mean and standard deviation.

### 2.4. Characterization of Degradation Behavior

The degradation morphology and microstructure of both groups was characterized by scanning electron microscopy (SEM-S4800, Hitachi, Japan) with an energy dispersive X-ray spectroscope (EDS, EMAX-7000, Horiba, Japan). To get a clean surface on the ends of the CGFs so the morphology could be analyzed in detail, the ends of the biocomposite pins were cut and then cleaned ultrasonically in deionized water before being dried. An analysis of a cross-section of the pins was also performed by first quenching the pins in liquid nitrogen and then manually breaking the pins to present an untarnished surface. 

The initial mass (*m_0_*) and dry mass (*m_dry_*) of the pins at each interval was weighed using a precision balance (BSA250S-CW, Sartorius). The change in mass of the pins was calculated by Equation (1).

Mass change (%) = m_dry_/m_0_ × 100 (1)

The inherent viscosity of the polymer matrix was measured using an automatic kinematic viscosity measuring system (VISCO 370, JULABO Labortechnik GmbH, Germany) to characterize the molecular weight. A special cutter (NETZSCH, Germany) was used to retrieve a mass of *m_v_* ± 0.2 mg (calculated by Equation (2)) from three pins in both groups. The mass obtained from each pin was dissolved separately in 30 mL of chloroform overnight. The volume of solution was then measured with a 50 mL volumetric flask before filtering out any inorganic residue with a 0.22 μm filter membrane. The final polymeric matrix solution had a concentration of 1 mg/mL. The total content of inorganic residue (*X_r_*, in %) from both pins was measured with a thermo-gravimetric analyzer (TGA 209F1, Netzsch-Gerätebau GmbH, Germany) from 50 °C to 600 °C with the temperature increasing by 10 °C/min. Measurements were taken in a nitrogen atmosphere and in an isothermal oxygen atmosphere for 30 min to burn up the residual organic carbon present in the PLDLA. The inherent viscosity (in mL/mg) at each interval (n = 3) was calculated from the sample’s flow time (*T_s_*, in s) and chloroform flow time (*T_c_*, in s) using Equation (3). Measurements were performed at 25 °C.

m_v_ = 50/(1−X_r_) (2)

(3) $$\mathrm{Inherent}\;\mathrm{viscosity}\;=\;\frac{\ln\frac{T_{s}-t_{s}}{T_{c}-t_{c}}}{1}$$

In Equation (3), t_s_ = sample Hagenbach-correction constant (in seconds) and t_c_ = chloroform Hagenbach-correction constant (in seconds).

The diameter of each pin was measured immediately after removal from the SBF. The diameters were measured with a slide caliper (Mitutoyo, 0–200 mm) and recorded in mm to an accuracy of two decimal places.

Differential scanning calorimetry (DSC 200F3, Netzsch-Gerätebau GmbH, Germany) was used to evaluate the thermal properties of the pins, including glass transition temperature (Tg), melting point (Tm), and melting enthalpy (ΔHf). Three samples were tested at each time point. The DSC measurements were conducted in a nitrogen (N_2_) atmosphere using the following heating steps: (i) from room temperature to −10 °C, (ii) from −10 °C to 200 °C at 10 °C min^−1^, (iii) from 200 °C to −10 °C at 10 °C min^−1^, (vi) isothermal for 15 min at −10 °C, (v) from −10 °C to 200 °C at 10 °C min^−1^, (vi) from 200 °C to room temperature at 40 °C min^−1^. The melting enthalpy (ΔHf) of 100% poly-L-lactide is 93.7 J/g [26]. The crystallinity of the polymeric matrix for both groups at different intervals was calculated by Equation (3) and the results have been presented as means and standard deviations.

Crystallinity (%) = ΔH_f_/93.7 × 100 (4)

### 2.5. Characterization of Calcium Phosphate Formation

Prior to analyzing the composition, the samples were first removed from the SBF and dried at room temperature under vacuum. The morphology and chemical composition of deposits on the surface of the pins after soaking in SBF was analyzed using SEM (S4800, Hitachi, Japan) with an EDS (EMAX-7000, Horiba, Japan). The chemical structure and composition of the deposition powder removed from the pins was characterized using X-ray diffractometry (XRD, D8 advance, Bruker, Germany) and Fourier-transform infrared spectroscopy (FT-IR; spectrum400, Perkin Elmer, MA, USA). 

## 3. Results

### 3.1. Degradation Behavior 

**Figure 1:**
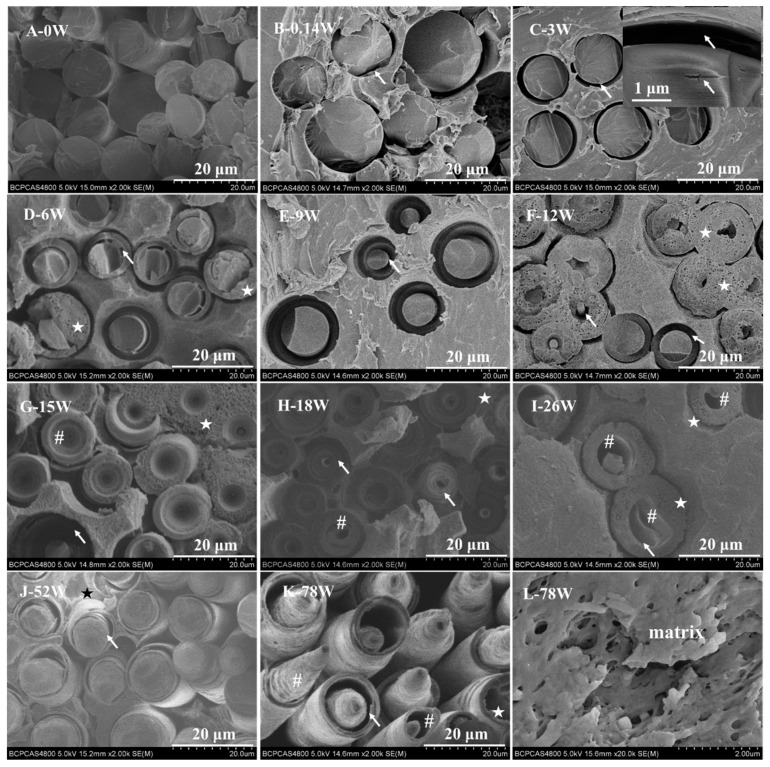
The end-face morphology of bioresorbable poly-_L_-co-_D_-lactide (PLDLA) composite pins reinforced with continuous glass fibers (CGFs) from 0 weeks to 78 weeks of degradation. Note: arrows indicate voids in the interface and cracks in the fibers; stars indicate deposits; hash signs indicate the funnel-shaped microstructure and microtubes.

The end-face micromorphology of some of the biocomposite pins is shown in Figure 1. A key finding was that the CGFs in the pins degraded through periodic spiral delamination. At 0.14 and three weeks, voids began to appear in the interface between the CGFs and polymeric matrix and cracks were apparent in the CGFs (Figure 1B,C). Between six and 12 weeks, the CGFs underwent a delaminating type of degradation, leading to the larger cracks, but the fibers remained largely intact (Figure 1D–F). Between 15 and 26 weeks, the delaminated CGFs gradually dissolved into the SBF and funnel-shaped microstructures and microtubes formed in the core of fibers, shortening their length (Figure 1G–I). The morphology at 52 and 78 weeks was similar to that at six and 12 weeks, signifying that the delamination must have ceased at some point between these times and then restarted as the time approached 52 weeks (Figure 1D–F,J–K). Mineral deposition was also observed on the end face of the biocomposite pins (Figure 1D–K, star marks) and by 78 weeks the polymeric matrix was noticeably more porous (Figure 1L). All pins were observed to have their structural integrity at each retrieved time point.

**Figure 2:**
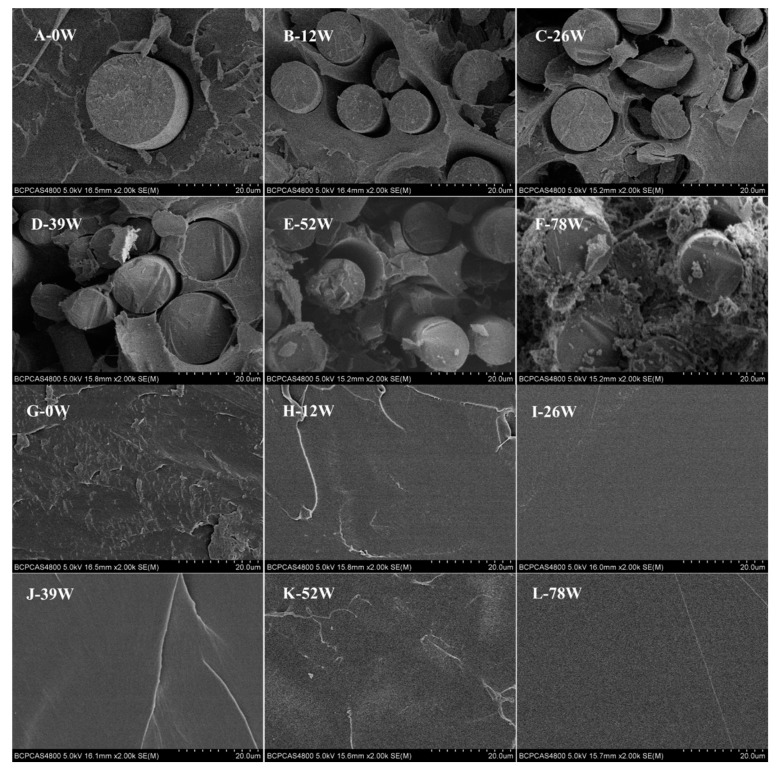
The cross-sectional morphology of bioresorbable PLDLA composite pins reinforced with CGFs (**A**–**F**) and pure PLDLA pins (**G**–**L**).

Figure 2 details the cross-sectional morphology of sample pins over a period of 78 weeks; (A–F) shows PLDLA composite pins reinforced with CGFs and (G–L) shows pure PLDLA pins. With degradation time, voids around the CGFs became larger, but whether there was any change in the diameter of the CGFs was not apparent and could not be detected due to the uneven diameter (Figure 2A–F). By 78 weeks the polymeric matrix of the reinforced pins was highly porous (Figure 2F), whereas there was very little change in the cross-sectional morphology of the pure PLDLA pins (Figure 2G–L).

**Figure 3:**
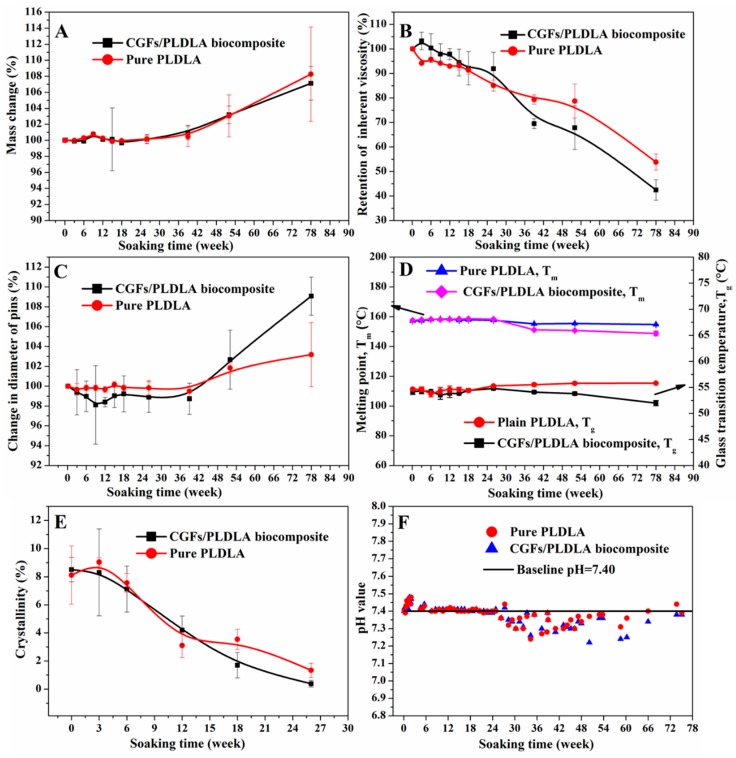
Material properties with degradation time.

The material properties of both groups of pins with increasing degradation time are shown in Figure 3. The increase in mass over time for both groups was due to mineral deposition, which indicated that the rate of deposition was higher than then rate of degradation (Figure 3A). The change in diameter over time for both groups was also due to this deposited surface layer, and the addition of CGFs accelerated the rate of deposition (Figure 3C). Figure 3B shows that while the retention of inherent viscosity decreased over time for both groups, the addition of CGFs to the polymeric matrix accelerated the decrease in viscosity. As the pins degraded, breaking of the polymer chains in both groups became evident by the decrease in Tg, Tm, and crystallinity (Figure 3D,E). The pH curve in Figure 3F also indicates rapid degradation of the biocomposite pins from 26 weeks (Figure 3F).

### 3.2. Calcium Phosphate Formation

**Figure 4:**
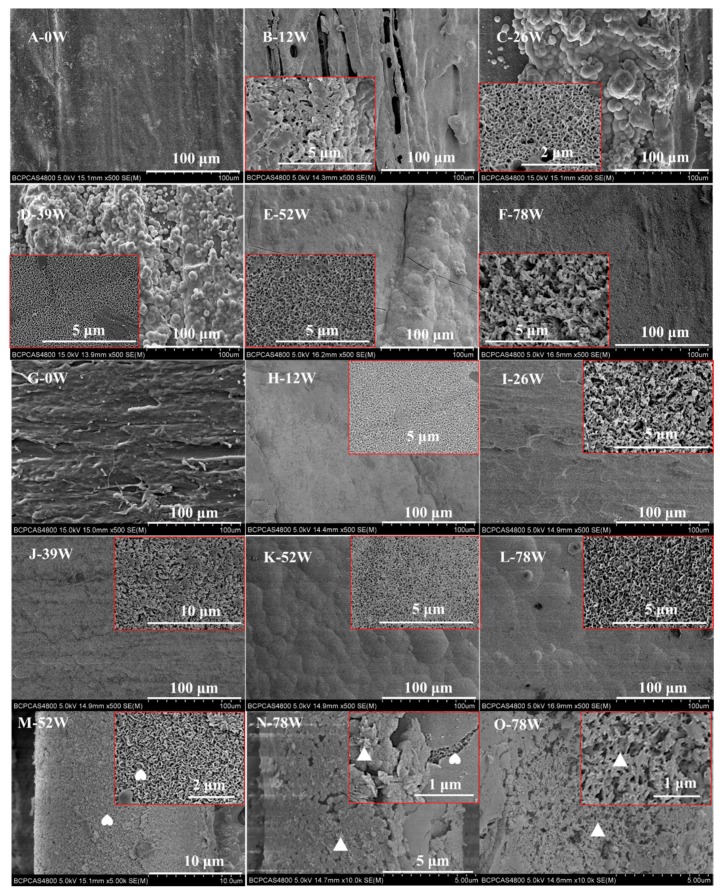
The surface morphology of PLDLA composite pins reinforced with bioresorbable CGFs (**A**–**F**), pure PLDLA pins (**G**–**L**), and CGFs (**M**–**O**). Note: magnified images on each image show the structure of the mineral deposits; the arrows indicate the surface of the CGFs; the rhombus indicates the surface of polymeric matrix; and the triangle and heart indicate the needle-like and sheet-like nanoapatite, respectively. Magnified images are presented in Figure A3 to clearly show the structure of the mineral deposits.

Figure 4 shows the morphology of deposits on the pins’ surface. The initial surface of both pins at week 0 was rough (Figure 4A,G) and the presence of CGFs embedded in the polymeric matrix was obvious (Figure 4A). In the biocomposite pins, needle-like (Figure 4B,F) and sheet-like (Figure 4C–E) nanocrystals firstly formed on the surface of CGFs, then deposited on the surface of the polymeric matrix, and finally formed an apatite layer. The two forms of nanocrystals are readily identifiable on the surface of the CGFs in Figure 4M,N. It can also be seen that the needle-like nanocrystals formed a textured cancellous bone-like layer (Figure 4O). By contrast, with the pure PLDLA pins, the sheet-like (Figure 4H,K,L) and needle-like (Figure 4I,J) nanocrystals formed an even layer on the pins’ surface. For both groups of pins, the apatite layer resembled the structure of bone (Figure 4B–L). 

**Figure 5:**
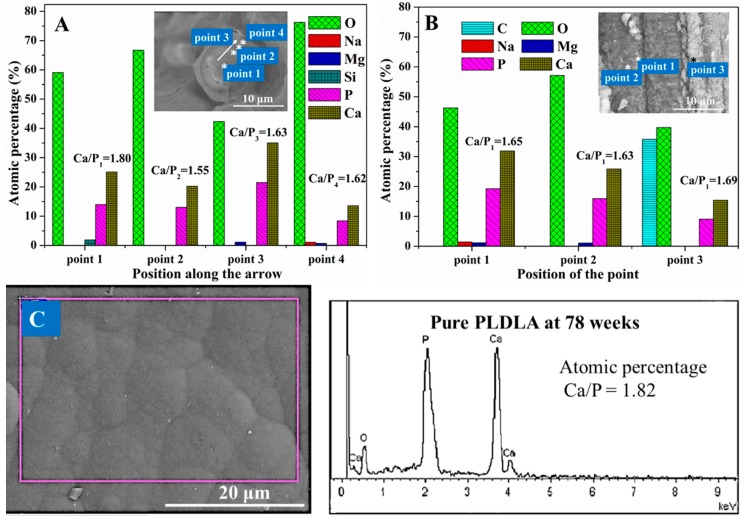
The composition of the end face (**A**) and surface (**B**) of biocomposite pins at 78 weeks; the composition of the surface (**C**) of pure PLDLA pins at 78 weeks.

The chemical composition of the mineral deposits on the end face of the CGFs (Figure 5A) and circumferential surface of the biocomposite pins (Figure 5B) and pure PLDLA pins (Figure 5C) at week 78 was analyzed by EDS. The analysis showed that the mineral deposits were primarily composed of oxygen, phosphorus, and calcium, with trace amounts of sodium and magnesium (Figure 5A,B). Figure 5A shows how the atomic ratio of calcium and phosphorous (Ca/P) changed from 1.80 to 1.62 on the end face of the CGFs, with the values decreasing in the direction of the white arrow. From Figure 5B it can be seen that at point 1 the nanosheet-like calcium phosphate contained trace amounts of sodium and magnesium and had a Ca/P ratio of 1.65; at point 2 the nanoneedle-like calcium phosphate contained trace amounts of magnesium and had a Ca/P ratio of 1.63; and at point 3 the calcium phosphate did not contain sodium or magnesium and had a Ca/P ratio of 1.69. For the pure PLDLA pins in Figure 5C, it can be seen that the Ca/P ratio of the nanosheet-like calcium phosphate on the surface was 1.82 at week 78. 

**Figure 6:**
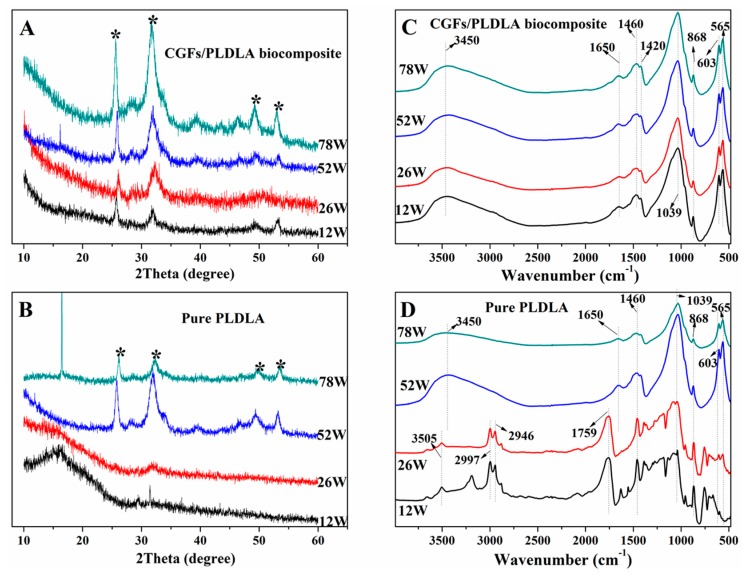
The X-ray diffraction (XRD) (**A,B**) and Fourier-transform infrared (FTIR) (**C,D**) spectrums of powder deposited on the surface of both pins. The stars indicated the crystal faces of HA according to the JCPDS No. 09-0432, which is (201), (112), (213), and (004), corresponding to the peaks at 2θ = 25.6°, 32.0°, 49.3°, and 53.0°.

The XRD analysis revealed that the material deposited on the surface of both pins from week 12 to 78 was mainly composed of hydroxyapatite (HA). The pronounced signals at 2θ = 25.6°, 32.0°, 49.3°, and 53.0° were characteristic peaks according to the XRD standard cards for HA (JCPDS No.09-0432). These four peaks correspond to the three crystal faces of HA, that is, (201), (112), (213), and (004) (Figure 6A). However, the intensity of the main peak (201) on the surface of the pure PLDLA pins was low before 26 weeks (Figure 6B). The HA on the surface of the biocomposite pins showed a higher level of crystallinity that the pure PLDLA pins due to the intensity of the peaks (Figure 6A,B).

The FTIR spectrum of the deposits on the surface of the biocomposite pins displayed absorption peaks at 3450, 1650, 1460, 1420, 1039, 868, 603, and 565 cm^−1^ from 12 to 78 weeks (Figure 6C). The absorption peaks at 3450 and 1650 cm^−1^ could be attributed to O–H vibrations of absorbable water in the mineral powder. The absorption band between 1400 and 1500 cm^−1^ for C–O asymmetric stretching vibration peak split to form double peaks (1420 and 1460 cm^−1^). It differs from the singlet peak of the infrared absorption spectrum of carbonate or free CO_3_^2−^ in this band, where the splitting of the absorption peak is a sign that CO_3_^2−^ has entered the HA lattice [27,28]. The peaks at 565 and 603 cm^−1^ are the in-plane bending vibration absorption peaks of PO_4_^3−^ in HA, 868 cm^−1^ is the symmetric stretching vibration absorption peak of PO_4_^3−^, and 1039 cm^−1^ is the asymmetric stretching vibration absorption peak of PO_4_^3−^ [29]. This indicates that the deposit is carbonate HA (CHA). Although the FTIR spectrum of the deposits on the surface of the pure PLDLA pins displays the same absorption peaks for C–O, CO_3_^2−^, and PO_4_^3−^ from 12 to 78 weeks, the peaks at 3505, 2997, 2946, and 1759 cm^−1^ are due to the O–H, C–H, and C=O stretching vibrations from residual polymeric matrix cut from the pure PLDLA pins [30] (Figure 6D). 

## 4. Discussion

The in vivo degradation behavior of medical devices can be estimated to a certain extent by analyzing their in vitro degradation [1,21]. The formation of a bone-like layer on the surface of a medical device is highly beneficial for encouraging bonding to bone [31,32,33,34]. Thus, the degradation behavior and apatite formation of bioresorbable PLDLA composite pins reinforced with CGFs were investigated in this study to evaluate the feasibility for use in load-bearing orthopedic applications. 

### 4.1. Degradation Behavior

Degradation behavior plays an important role in the success of resorbable implants for use in load-bearing applications. In addition to the effects on mechanical performance, the degradation mode can have a substantial effect on long-term clinical benefits [35]; fragmentation degradation of pure polylactide has been shown to result in late complications [36] and rapid delamination of phosphate glass fibers (PGFs) can lead to a mismatch with the rate of bone ingrowth [37,38].

**Figure A1:**
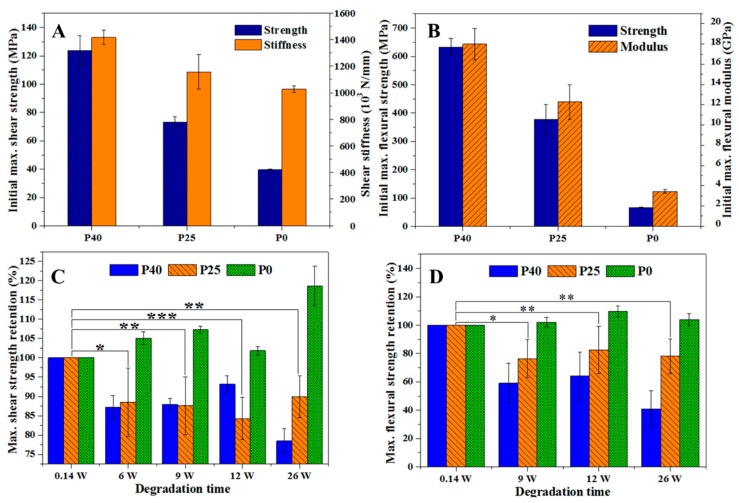
(**A**) The initial maximum shear strength and stiffness of P40, P25, and P0 materials; (**B**) the initial maximum flexural strength and modulus of P40, P25, and P0; (**C**) shear strength retention with degradation time; and (**D**) flexural strength retention with degradation time. The statistical analysis of the shear and flexural strengths was shown in Figure A1C-D, in which P < 0.05 was considered statistically significant; * P < 0.05 termed significant level 1; ** P < 0.01 termed significant level 2; *** P < 0.005 termed significant level 3.

**Figure A2:**
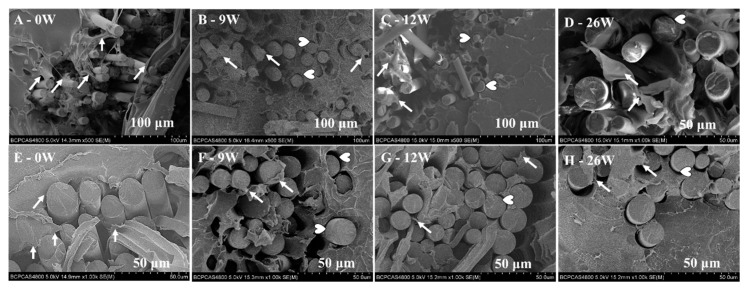
Scanning electron microscopy (SEM) images showing the pull-out effect of CGFs in the biocomposite pins (P25) from 0 weeks to 26 weeks during (**A**–**D**) flexural testing and (**E**–**H**) shear testing. The white arrows indicate strong bonding between the fibers and matrix and the heart-shaped markers show voids at the interphase boundary.

A significant finding of this work was the periodic degradation of the end face of the biocomposite pins which resulted in compelling nano- and micro-structures (Figure 1). The periodic degradation occurred in four steps: (1) nano-cracks appeared around the fibers at the matrix interface; (2) a spiral delaminating type of degradation occurred on the core of the CGFs; (3) funnel-shaped and micro-tube structures appeared at the center of the CGFs; and (4) the ends of the CGFs eroded, shortening their length, and the polymer matrix became much more porous. The formation of nano-cracks was due to the dissolution of CGFs in the SBF in step 1. The capillary effect of these cracks led to more water being diffused into the matrix interphase [1], which subsequently led to the formation of a hydrated layer on the surface or near the surface of the CGFs, resulting in a spiral delaminating type of degradation in step 2. As the CGFs underwent delamination the interphase boundary gradually weakened (Figure 1 and Figure 2A–F), which presented as a pull-out effect during mechanical testing (Figure A2). Also, the shear and flexural strength of the biocomposite pins gradually decreased over time as the materials degraded (Figure A1). Previous studies have reported that delamination of PGFs [37,38,39,40,41] occurs due to the formation of hydrated outer layers as a consequence of their dissolution mechanism. This is a similar mechanism to the delamination of silicate glass fibers whereby the outer layers undergo hydration reactions and breakage of the glass network [41]. However, in this study, a dramatically different spiral delamination mode occurred which resulted in the formation of funnel-shaped and micro-tube structures, as identified in step 3 above. It has been speculated that the delamination relates to the processing technique [38] and chemical composition [37] of the fibers. The funnel-shaped and micro-tube structures formed due to bulk degradation of the CGFs, where the CGFs degraded internally from their cores and the hydrated layer did not leach into the surrounding medium, leaving it to act as a protective layer. Finally, as noted in step 4 above, the fibers shortened due to water molecules penetrating through the capillary-like channels that formed as the fibers degraded [37]. Moreover, the capillary-like channels and micro-tubules also improved the migration rate of and degradation byproducts of the polymeric matrix, acting to inhibit core-accelerated bulk degradation [1,21]. 

The CGFs located in the bulk of the biocomposite pins did not undergo delamination until week 78 (Figure 2A–F). However, prior to the onset of delamination, water had diffused into the pins which caused more voids to form around the CGFs. The presence of CGFs accelerated the degradation rate of the pins (Figure 2F,L) as a result of the interaction between the fibers and the polymer matrix whereby the hydroxyl ions attacked the ester groups in the matrix [21].

This study has revealed that the addition of CGFs increased the degradation rate of pure PLDLA and increased the amount of apatite deposits on the surface during the degradation process (Figure 3). The accelerated degradation was due to the interaction of alkali and acidic byproducts in the CGFs and polymeric matrix [21]. The change in mass of the biocomposite pins was a result of polymer degradation, fiber dissolution, and apatite precipitation (Figure 3A); apatite precipitation was the main contributor to the increased mass seen for both materials. The apatite precipitation was also a contributor towards the increased diameter of both pins (Figure 3C) [14]. Due to the higher degradation rate of the biocomposite pins, their thermal properties, such as Tg, Tm, and crystallinity, were slightly lower than those of the pure PLDLA. The lower thermal properties corresponded to the difference in inherent viscosity between the pins. Thus, the content of CGFs could be altered in order to tailor the degradation rate of PLDLA for use in different load-bearing implants.

In comparison to PGFs [37], the CGFs in this study underwent a drastically different form of spiral delamination and had a much lower degradation rate. This could improve the long-term strength retention of implants (Appendix Figure A1 and Figure A2), making these materials useful for load-bearing applications.

### 4.2. Calcium Phosphate Formation

The ability of a material to form apatite in SBF is widely used to evaluate its bioactivity. An apatite layer deposited on the surface of a bioactive implant needs to demonstrate the following properties according to ISO 23317, which are similar to the properties of bone mineral: (1) being calcium deficient; (2) having a lower Ca/P atomic ratio than stoichiometric apatite; 3) containing impurities such as Mg^2+^, Na^+^, Cl^−^, and HCO_3_^−^; (4) having low crystallinity. 

In this current study, an apatite-rich layer formed on the surface of both materials (Figure 4). When the biocomposite pins were immersed in SBF there was clear precipitation of HA on the CGFs, which was due to the following reactions [1]: (1) the acidic degradation of the polymeric matrix producing HA byproducts; (2) the dissolution of CGFs, whereby the alkali and alkali earth metals reacted with water to form basic hydroxides in the interphase resulting in a high local pH; (3) the neutralization of the products in (1) and (2); and (4) deposition of a Ca-P layer on the silicate-rich surface layer of the CGFs (Figure 5A). 

The calcium phosphate on the surface of both pins produced nano-sheet and nano-needle apatite structures (Figure 1, Figure 4 and Figure 5), which is similar to the structure of bone mineral [42]. Previous studies [5,43,44] have demonstrated that the quantity and appearance of calcium phosphate crystals depends on the soaking medium, soaking time, and matrix. This aspect may be investigated in future studies. 

The surface deposits were composed of carbonated HA doped with sodium and magnesium ions (Na^+^ and Mg^2+^), which was confirmed by XRD, FTIR, and EDS analysis (Figure 4 and Figure 5). Due to the carbonate (CO_3_^2−^), the Na^+^ and Mg^2+^ exchanged with the Ca^2+^ within the network of apatite (Figure 5), leading to a calcium-deficient deposit of apatite. Magnesium is a minor but important component of bone, enamel, and dentine, and it plays an essential role in the bio-mineralization process [45,46]. Compared with pure HA of the same crystallinity, Mg-doped HA implant materials offer better adhesion and proliferation of osteoblast cells [36]. Therefore, a HA surface can layer presents superior osteoconductivity, can promote cellular function, and offers excellent biocompatibility [47,48]. The apatite layer deposited on the biocomposite pins investigated in this study had a lower Ca/P ratio than the stoichiometric HA (1.67) (Figure 5A,B), whereas the Ca/P ratio of the apatite layer deposited on the pure PLDLA was 1.82 (Figure 5C). This indicated that the addition of CGFs enhanced the bioactivity of PLDLA in vitro. According to the broad peaks of 2θ = 32.0° in the XRD spectrum and 1039 cm^−1^ in the FTIR spectrum (Figure 6), the low degree of splitting indicated the presence of apatite with low crystallinity [29]. Thus, the apatite deposited on the biocomposite pins had similar characteristics to bone mineral. 

The results of this study strongly support the use of these novel CGF-reinforced biocomposite pins in load-bearing orthopedic applications. Future studies may investigate the mechanism behind the formation of different crystal nanostructures on the surface of the biocomposite pins. This may relate to the selection of matrix type (altering the degradation rate and acidic byproducts), content of bioresorbable glass fibers (composition, processing method, and alkali byproducts) and soaking medium (prepared and controlled method). However, two of the key properties of the biocomposite pins demonstrated in this study that support their use for load-bearing orthopedic applications are their spiral delamination degradation mode and high bioactivity in vitro. 

Related to the section above on how this research may be expanded in the future, there are some limitations with the methods used in this study that should be noted: (1) the study mainly focused on the degradation of CGFs in a PLDLA matrix, but did not analyze how different materials for the matrix may impact the degradation behavior. However, the degradation features of the PLDLA (L/D = 80/20) matrix in this study were shown to be comparable to matrices composed of different materials from the literature [1]. This demonstrates the reliability of the results presented in this study and the practicality of the implants for load-bearing applications. (2) the implants analyzed in this study had a basic pin shape. More complex implants (screw thread, pores, or hollow core, etc.) may be considered in future studies. (3) A constraint of this study is that the effect of cell-related mechanisms, such as cytocompatibility in vitro and histological response in animal testing, was not considered [1]. Studies have shown that the composition, structure, crystallinity, and degradation rate of bioglass fibers and mineralized HA are key factors that affect the proliferation and differentiation of osteoblasts [18,25,49]. Therefore, future work may firstly consider the cytocompatibility of such reinforced bioresorbable composites reinforced with high strength CGFs.

## 5. Conclusions

In this work, the spiral delamination of the end face of CGFs acted to slow the degradation rate of biocomposite pins. Implant materials with a simple structure, such as the PLDLA-CGF implant investigated in this study, degrade from each end towards the center, and from surface degradation to bulk degradation. The degradation behavior is crucial for the success of any implantable material, particularly in load-bearing applications. This study has demonstrated that the apatite deposited on biocomposite pins is similar to bone mineral, confirming the bioactivity of the implant and making it potentially useful for load-bearing orthopedic applications.

## Figures and Tables

**Table 1:** Ion concentrations of simulated body fluid (SBF) and human blood plasma according to ISO 23317.

Ion	Concentration (10^−3^ mol/L)	
	SBF (pH 7.40)	Blood Plasma (pH 7.2 to 7.4)
Na^+^	142.0	142.0
K^+^	5.0	5.0
Mg^2+^	1.5	1.5
Ca^2+^	2.5	2.5
Cl^−^	147.8	103.0
HCO_3_^−^	4.2	27.0
HPO_4_^2−^	1.0	1.0
SO_4_^2−^	0.5	0.5

**Figure A3:**
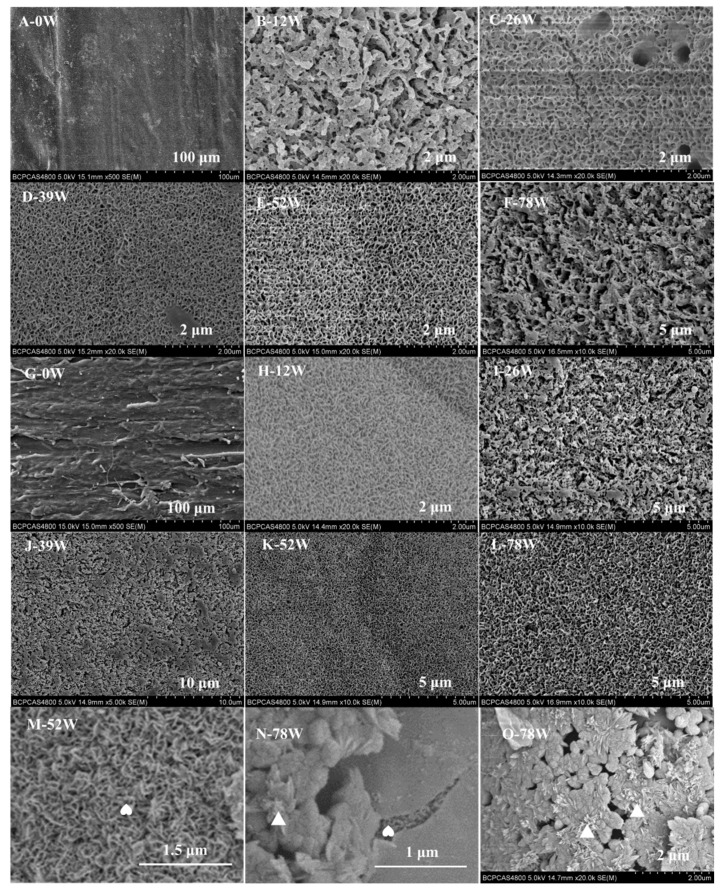
Magnified morphology of nanoapatite on the surface of PLDLA composite pins reinforced with bioresorbable CGFs (**A**–**F**), pure PLDLA pins (**G**–**L**) and CGFs (**M**–**O**). The triangles and hearts indicate the needle-like and sheet-like nanoapatite, respectively.

## Appendix A

In our previous study [50], the effects of varying the content of bioresorbable CGFs on the mechanical behavior of biocomposite pins were investigated. The results showed that changing the content of glass fibers in the biocomposites had a considerable effect on the initial mechanical properties and strength retention over the degradation time (Figure A1). A pull-out effect of the CGFs from the matrix was observed during mechanical testing (Figure A2). The shear and flexural strength of the biocomposite pins gradually decreased as the materials degraded (Figure A1). The P25 pins, which maintained the sufficient initial mechanical properties and mechanical retention timeframe for at least 26 weeks, have excellent potential for orthopedic applications in load-bearing sites.

P40, P25, and P0 indicated biocomposite pins with 40 wt %, 25 wt %, and 0 wt % content of CGFs.

Additional images magnified from Figure 4 are shown in Figure A3.
